# Physicochemical Characterization of Resistant Starch Type-III (RS3) Obtained by Autoclaving Malanga (*Xanthosoma sagittifolium*) Flour and Corn Starch

**DOI:** 10.3390/molecules26134006

**Published:** 2021-06-30

**Authors:** Vicente Espinosa-Solis, Paul Baruk Zamudio-Flores, Miguel Espino-Díaz, Gilber Vela-Gutiérrez, J. Rodolfo Rendón-Villalobos, María Hernández-González, Francisco Hernández-Centeno, Hayde Yajaira López-De la Peña, René Salgado-Delgado, Adalberto Ortega-Ortega

**Affiliations:** 1 Coordinación Académica Región Huasteca Sur, Universidad Autónoma de San Luis Potosí. Km 5, Carretera Tamazunchale-San Martín, Tamazunchale, San Luis Potosí C.P. 79960, Mexico; vicente.espinosa@uaslp.mx; 2 Centro de Investigación en Alimentación y Desarrollo, A.C. Unidad Cuauhtémoc, Fisiología y Tecnología de Alimentos de la Zona Templada, Avenida Rio Conchos s/n, Parque Industrial, Apartado postal 781, Ciudad Cuauhtémoc, Chihuahua C.P. 31570, Mexico; aster3000@hotmail.com; 3 Laboratorio de Investigación y Desarrollo de Productos Funcionales, Facultad de Ciencias de la Nutrición y Alimentos, Universidad de Ciencias y Artes de Chiapas, Libramiento Norte Poniente 1150, Col. Lajas Maciel, Tuxtla Gutiérrez, Chiapas C.P. 29000, Mexico; gilber.vela@unicach.mx; 4 Centro de Desarrollo de Productos Bióticos, Instituto Politécnico Nacional, Calle Ceprobi No. 8, Colonia San Isidro, Yautepec, Morelos C.P. 62731, Mexico; rrendon@ipn.mx; 5 Departamento de Ciencia y Tecnología de Alimentos, División de Ciencia Animal, Universidad Autónoma Agraria Antonio Narro, Calzada Antonio Narro 1923, Buenavista, Saltillo, Coahuila C.P. 23515, Mexico; maryhg12@yahoo.com (M.H.-G.); franciscohdezc@gmail.com (F.H.-C.); yajaira.lp@gmail.com (H.Y.L.-D.l.P.); 6 Tecnológico Nacional de México/Instituto Tecnológico de Zacatepec, Posgrado-Departamento de Ingeniería Química y Bioquímica, Calzada Tecnológico 27, Zacatepec, Morelos C.P. 62780, Mexico; rene.sd@zacatepec.tecnm.mx; 7 Facultad de Ciencias Agrotecnológicas, Universidad Autónoma de Chihuahua, Extensión Cuauhtémoc, Barrio de la Presa s/n, Ciudad Cuauhtémoc, Chihuahua C.P. 31510, Mexico; aortega@uach.mx

**Keywords:** malanga flour, FTIR spectroscopy, resistant starch, amylose molecular weight, color evaluation

## Abstract

The feasibility of obtaining resistant starch type III (RS3) from malanga flour (*Xanthosoma sagittifolium*), as an unconventional source of starch, was evaluated using the hydrothermal treatment of autoclaving. The physicochemical characterization of RS3 made from malanga flour was carried out through the evaluation of the chemical composition, color attributes, and thermal properties. In addition, the contents of the total starch, available starch, resistant starch, and retrograded resistant starch were determined by in vitro enzymatic tests. A commercial corn starch sample was used to produce RS3 and utilized to compare all of the analyses. The results showed that native malanga flour behaved differently in most of the evaluations performed, compared to the commercial corn starch. These results could be explained by the presence of minor components that could interfere with the physicochemical and functional properties of the flour; however, the RS3 samples obtained from malanga flour and corn starch were similar in their thermal and morphological features, which may be related to their similarities in the content and molecular weight of amylose, in both of the samples. Furthermore, the yields for obtaining the autoclaved powders from corn starch and malanga flour were similar (≈89%), which showed that the malanga flour is an attractive raw material for obtaining RS3 with adequate yields, to be considered in the subsequent research.

## 1. Introduction

Malanga (*Xanthosoma sagittifolium* L. Schott) is the common name for one of the most popular and widely consumed aroid cultivars. The other names for this corm include yautia, taioba, tanni, or new cocoyam, depending on the country of origin [1,2]. Malanga is an important food crop in tropical and subtropical regions, and is consumed mainly cooked or in the form of pasta, similarly to potatoes [3,4]. Malanga corms contain up to ≈28% of starch, making them a significant, middle-range source of dietary energy [5]. Malanga flour forms good quality pasta, with great potential for application in the food industry [6]; because of its starch content, non-starchy polysaccharides, proteins, vitamins, minerals, and total dietary fiber, malanga flour could be considered as a functional food [1,7]. Malanga flour contains up to 4.21% resistant starch (RS) [1], which is probably starch molecules that form complexes with proteins. Malanga starch granules have a typical A-type X-ray diffraction pattern, with amylopectin containing a high proportion of shorter branched chains that cause lower structural stability, making the starch more digestible [8].

Most of the native starch is completely digested and absorbed in the small intestine; however, a small fraction of the starch, known as resistant starch (RS), cannot be hydrolyzed to free glucose, since it resists the hydrolytic attack of digestive enzymes [9]. RS arrives intact to the large intestine, where it is fermented by the microflora of the colon, resulting in the production of short-chain fatty acids, such as acetic, propionic, and butyric acids, CO_2_, H_2_, and in some individual CH_4_; this causes increased fecal bulk and lower colonic pH. Thus, RS decreases colon cancer risk [10,11]. RS is classified into the following four types: RS1, which is physically inaccessible in plant tissue because it is trapped in a matrix of proteins or cellulose; RS2 is a native starch in the granules, and is resistant by its semi-crystalline character; RS3, which is a retrograded starch, produced by physical heat-humidity and cooling temperature treatments (*v*. *gr*. the application of autoclaving treatments); and RS4, which is obtained by the chemical modification of native starch [11,12]. RS is considered a functional food, since it can prevent cardiovascular diseases, colon cancer, diabetes, obesity, osteoporosis, and it improves intestinal health, micronutrient absorption, lipid profile, satiety, and the development of probiotic microorganisms [10,12,13]. Added to baked products, such as cakes and muffins, the RS improves the final product’s texture without affecting its sensory properties [14]. RS3 does not require chemical compounds for obtaining it, which makes it a safe food. It can be formed during hydrothermal or lyophilized processing, and by retrogradation of gelatinized starch or temperature cycles during cooking and cooling [15,16,17,18,19].

Several studies have been published on obtaining resistant starch type-III (RS3) from corms and tubers starches, such as taro starch [20], purple yam [21], and potato starch [22]. However, no studies have been reported that enhance the functionality of corm flour by direct autoclaving it to obtain RS3, or the effect of other components of the flour on the formation of RS3. Therefore, this study aimed to compare the physicochemical properties, chemical composition, morphology, color attributes, thermal properties, starch digestibility, and structural features between malanga flour, corn starch, and their respective resistant starches that are obtained by autoclaving treatment.

## 2. Results and Discussion

### 2.1. Chemical Composition

The chemical composition of malanga flour (MF), corn starch (CS), and their respective resistant type III starches (autoclaved malanga flour (AMF) and autoclaved corn starch (ACS)) are shown in Table 1. All of the samples showed slight differences regarding the ash, moisture, and carbohydrate contents, while significant differences were observed (*p* < 0.05) in the protein, lipid, and total dietary fiber contents. MF had a protein content (≈2.8%) that was 1.5, 3, and 7 times greater than AMF, CS, and ACS, respectively. Cocoyam flours have been reported as a poor source of protein, with values ranging from 7.5 to 2.1% [2,23].

The moisture contents of MF (15.65%) and CS (14.36%) were higher than other studies reported for malanga flours (8.87 to 10.67%) [7], and malanga and taro starches (13.43 and 14.01%) [24]. The above suggests that this parameter depends on the botanical source, the plant tissue used, the storage conditions of the flours [25,26], and the moisture absorption capacity of the flours and starches [26,27,28]. In addition, the ash content of cocoyam flours is directly related to the content of minerals, such as phosphorus, zinc, iron, and calcium (as calcium oxalate crystals) [29].

MF and AMF showed no significant differences (*p* > 0.05) in the ash content, although the ash content values were high (6%), suggesting that they may have significant amounts of the above elements. The phosphorus content is important in the functional properties of starches, and their content is mainly attributed to the presence of ester phosphate groups [24,30]. On the other hand, the ACS sample presented a very low ash content, although the content of chemical and mineral compounds depends not only on the botanical source, but on the extraction methods used. For example, in the scientific literature, it has been reported that the autoclaving process reduces the ash content, due the high pressures and high temperature that cause the breaking of the ester bonds of the phosphate groups, with a consequent reduction in the ash content [16,24,31].

**Figure 1:**
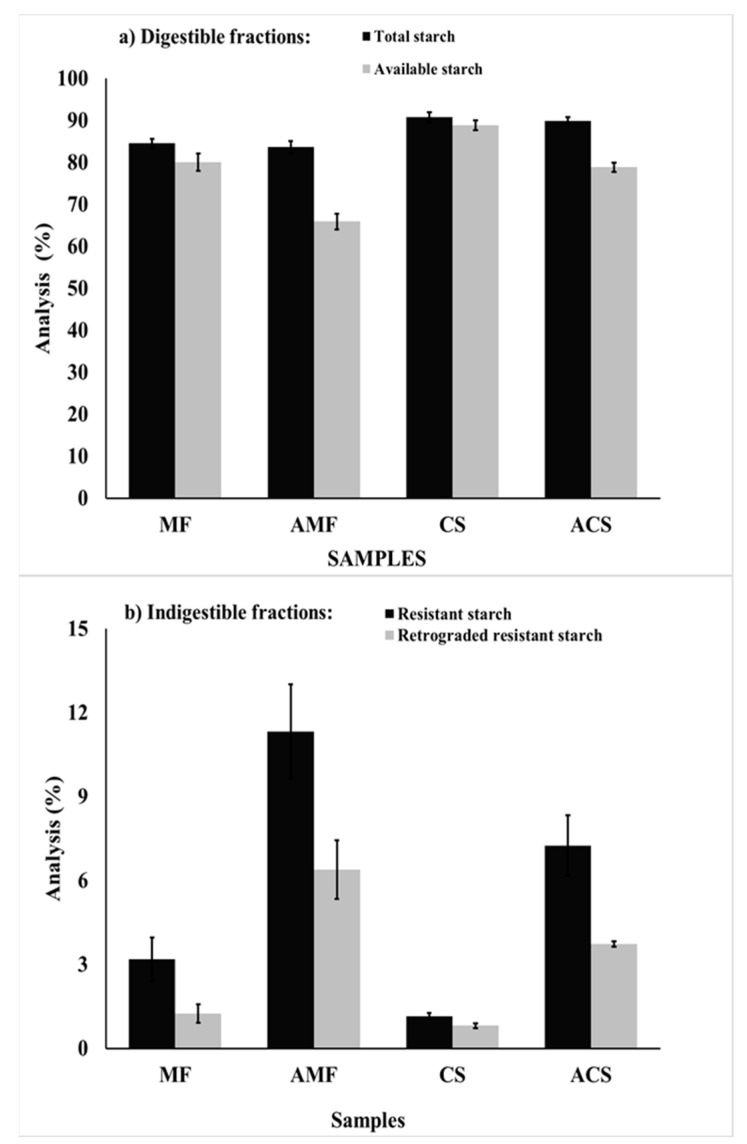
Results of the (**a**) digestible and (**b**) indigestible starch fractions of malanga flour (MF), autoclaved malanga flour (AMF), corn starch (CS), and autoclaved corn starch (ACS). Results are average from at least three repetitions ± standard error bars.

The major component of MF, AMF, CS, and ACS powders were carbohydrates expressed as total carbohydrates (Table 1), mainly constituted by starch (Figure 1a) and fiber. As shown in Table 1, CS and MF had higher protein and total dietary fiber contents than their autoclaved counterpart powders, although this did not occur with the lipid and carbohydrate molecules. Therefore, it is assumed that autoclaving treatment produces the denaturation and solubilization of proteins, besides it causes the depolymerization of fiber polysaccharides and the subsequent leaching of the components of dietary fiber, thus increasing the total carbohydrate content after the autoclave process. Finally, the yield for obtaining derived powders of CS and MF was 89% in both cases. This percentage indicates that the autoclaving–cooling treatment of malanga flour is a suitable treatment to obtain resistant starch type III, with moderate material losses.

### 2.2. Granule Morphology

**Figure 2:**
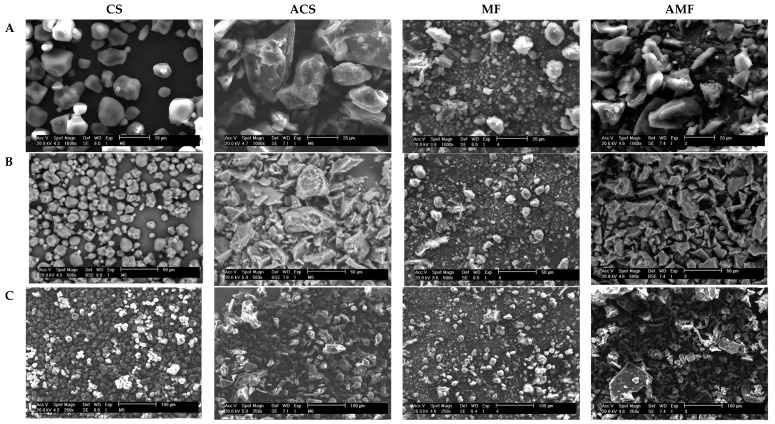
Micrographs of corn starch (CS), malanga flour (MF), autoclaved corn starch (ACS), and autoclaved malanga flour (AMF). Analyzed at 1000× magnification (**A**), 500× magnifications (**B**), and 250× magnifications (**C**).

The images obtained by the scanning electron microscopy (SEM) of the samples are presented in Figure 2. The CS granules had a typical morphology of normal maize starches, with spherical, elliptical, and polyhedral granules and granule fragments, because of the grinding to obtain starch. The morphology of the CS granules coincides with that reported by other studies of corn starch granules [32,33,34]. The micrograph of MF (Figure 2) showed small and rounded starch granules and other large ellipsoidal granules, similar to those reported for *Xanthosoma* sp. [3,24,35,36]. Figure 2 shows the presence of other components of fiber in the malanga flour, which presented the greatest amount of fiber (Table 1). The autoclave treatment caused the gelatinization and destruction of the native structural arrangement of the starch granules in both the ACS and AMF. This hydrothermal treatment (autoclaving) also significantly reduced the molecular weight and apparent amylose content, which is consistent with the values of these variables observed in Table 2. This effect has been reported in other works of obtaining RS by autoclaving starches of other plant species, such as corn and oats [10,37,38].

### 2.3. In Vitro Digestibility of Native and Autoclaved Samples

The results of the total starch (TS), available starch (AS), resistant starch (RS), and retrograded resistant starch (RRS) are shown in Figure 1. The techniques of TS, AS, RS, and RRS are known as in vitro enzyme digestibility tests of starch, and their determinations are approximate quantifications based on models that emulate gastrointestinal conditions, so they represent an approach to in vivo studies [39,40,41]. Starch was the main molecule presented in all of the samples, MF showed 84.55% of TS, which is higher than the results reported by Markusse et al. [7] for cocoyam flours (*Xanthosoma sagittifolium*) from white and red flesh, which was 63.47 and 69.16%, respectively, and similar to taro flour (*Colocasia esculenta* L. Schott) from different varieties, ranging from 82.1 to 86.6% [25]. There was no significant effect (*p* > 0.05) on the TS content of MF and CS samples after the autoclave treatment, while the AS content decreased at 14.16 and 9.96% in MF and CS, respectively. The apparent amylose content and the average amylose molecular weight (Table 2) were significantly reduced (*p* < 0.05) by the hydrothermal treatment. The reduction in amylose molecular weight is consistent with the decrease in AS content (Figure 1a) and the increment of RS content (Figure 1b). The different values of AS and RS among the samples may be because of factors such as the botanical source of the native starch, the amylose-–amylopectin ratio, branching length in the amylopectin, percentage of crystallinity, formation of the amylose–lipid complex, and pores or fractures within the granules [12,15,38,42].

The indigestible fractions of starch (RS and RRS) are shown in Figure 1b. The ACS and AMF samples had a higher RS and RRS content than their native counterparts. AMF presented 2.5 and 4 times more RS and RRS than MF, which shows that autoclaving is efficient in the formation of RS. The increased content of RRS can be attributed to the recrystallization of amylose during cooling, by forming densely compact structures that are stabilized by hydrogen bonds [10], and to the formation of the amylose–lipid complex, which is resistant to the breakage of digestive enzymes [43].

### 2.4. Physicochemical Parameters

#### Color Attributes

The color attributes lightness (L*), chroma (C*), and hue angle (°h) of MF, CS and their respective autoclaved powders are shown in Table 3. Except for the AMF, the samples showed a high luminosity value (L* > 90). The values of °h of MF (≈39) and AMF (≈65) indicate that the malanga flour presented a reddish color, which slightly decreased after the autoclaved treatment of malanga flour, deviating towards a yellow °h; an increase in chroma (C*) value, from 4.49 to 14.26, showed a higher color saturation after the autoclaved treatment. The L* value of MF (≈92) was reduced in the AMF (≈78). The variations in lightness and °h of MF and CS could be related to pigments, such as carotenes and xanthophylls, present in the MF, which impart a slightly reddish °h to the flour [3], which could be reduced by hydrothermal treatment. In the CS and ACS samples, the same trends were observed, but with minimal changes L* (100 to 97), C* (4.9 to 2.7), and °h (106 to 100), as shown in Table 3. In these samples, the °h values showed a less reddish hue, closer to yellow, than in the malanga samples. Autoclaving to obtain the resistant powders affected both the CS and MF; during this process, the protein and carbohydrate content present in the samples (Table 1) could have generated non-enzymatic darkening reactions (Maillard reactions), which occur between the amino groups of the proteins and the carbonyl groups of the reducing sugars, or as the final product of lipid oxidation [44].

### 2.5. Thermal Properties

Differential scanning calorimetry (DSC) is used to monitor changes in thermal energy, associated with physical and chemical transformations of materials, as a function of temperature. The results of the thermal analysis of the samples are presented in Table 4. The MF presented higher values for the onset temperature (T_o_ = 75.14 °C), peak temperature (T_p_ = 84.56 °C), conclusion temperature (T_c_ = 92.03 °C), and enthalpy change (ΔH = 12.49 J/g) of gelatinization, compared to the CS sample (Table 4). The T_p_ value of MF is consistent with those values reported by Perez et al. [29] for cocoyam flours (T_p_ = 85.5–90.3 °C), and Hoyos-Leyva et al. [2] for starches from *Colocasia esculenta* and *Xanthosoma sagittifolium* varieties (T_p_ = 80.6–84.8 °C). Table 4 shows that the autoclaved samples (ACS and AMF) did not show significant differences (*p* > 0.05) between them in any of the thermal variables analyzed, but they presented higher values of T_o_, T_p_ and T_c_ compared to their corresponding native sample, CS and MF, respectively. The results indicate that these native samples, when subjected to the hydrothermal process of autoclaving with high pressure, apparently lose their native granular structure, giving rise to structures with greater thermal stability. Shah et al. [38] reported dual autoclaving–cooling cycles of oat starches. During this process, the granule structure was disrupted, gelatinized, and rearranged, leading to an increase in the T_o_, T_p_ and T_c_ parameters.

### 2.6. Fourier Transform Infrared (FTIR) Spectroscopy

**Figure 3:**
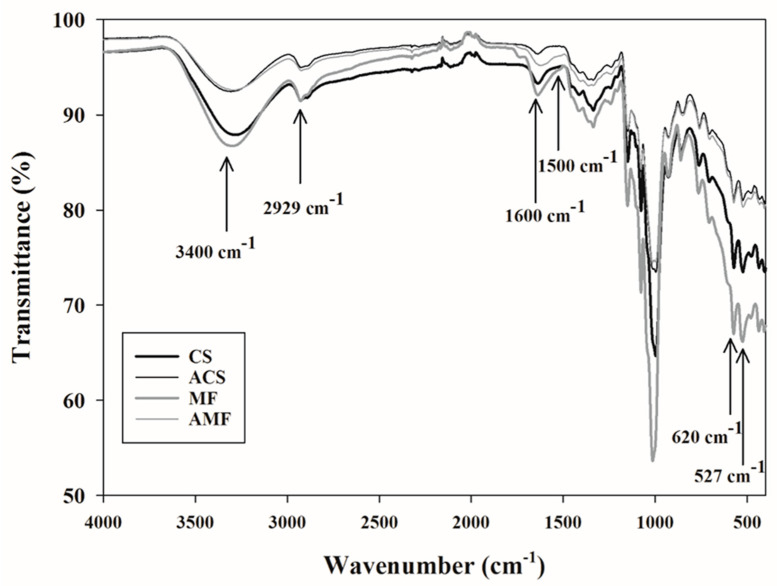
Fourier transform infrared (FTIR) spectra of samples. CS = corn starch; MF = malanga flour; ACS = autoclaved corn starch; AMF = autoclaved malanga flour.

The FTIR analysis performed on the samples is shown in Figure 3, which shows some bands and peaks that are characteristic of starchy carbohydrates. The samples CS, MF, and AMF displayed transmittance peaks in the wavenumbers (*n* = 1/λ) of 3400, 2929, 1500, 1600 and 620–527 cm^−1^, which correspond to the functional groups of poly-OH, -CH_2_, C–O–C (skeletal vibrational mode of the α-1,4 glucosidic linkage), carboxylate ion (COO^−^), and skeletal modes of the pyranose ring structure, respectively [12,15,38]. At the same time, the ACS sample showed most of the bands mentioned above, except for the band at 1500 cm^−1^, which evidenced the breakdown of the glycosidic bond caused by the high temperature and pressure of the autoclave treatment.

Figure 3 shows similarities between the CS and MF samples, with a higher intensity of some peaks (at 620 and 527 cm^−1^) in MF, probably due to other minor components, mainly fibers and some plant pigments. The samples submitted to the autoclaved treatment, ACS and AMF, showed similarities in the position of some characteristic peaks. The differences between the sources of RS3 that are consist with an extension of the peaks located at 3400 cm^−1^ suggest an increase in the crystalline region, as reported by Garcia-Rosas et al. [17] and Shah et al. [38].

## 3. Materials and Methods

### 3.1. Materials

Commercial corn starch (Maizena™, Unilever, Mexico) was acquired at a local market (Cuauhtemoc City, Chihuahua, Mexico). Malanga flour (*Xanthosoma sagittifolium*) was obtained using the dehydration of corms from plants cultivated in the municipality of San Fernando (Chiapas, Mexico). Corms were washed, disinfected with water and soap, and their shells were removed and cut into slices with a 5 mm thickness. The slices were placed in a 5% citric acid solution for 15 min, subsequently placed in trays, and underwent a conventional drying process for eight hours at 60 °C. The dried slices were ground and sifted with a mesh number 80, and the flour obtained was vacuum packed. All the chemical reagents used were analytical grade and were acquired from Sigma-Aldrich (Toluca, State of Mexico).

### 3.2. Preparation of Autoclaved Powders

An autoclaving–cooling temperature treatment physically modified native malanga flour (MF) and corn starch (CS) to obtain autoclaved malanga flour (AMF) and autoclaved corn starch (ACS) powders rich in retrograded resistant starch (RRS), according to the method used by Berry [45]. First, a dispersion was prepared by mixing 60 g of sample with 210 mL of distilled water. The mixture was autoclaved at 121 °C for 1 h with a pressure of 103 kPa. Once cooled, the dispersion was stored at 4 °C for 24 h and subsequently subjected to freeze-drying, grinding, and sieving processes. This methodology was conducted in triplicate.

### 3.3. Physicochemical Properties of Malanga Flour, Corn Starch and Their Respective Resistant Starches

#### 3.3.1. Chemical Composition

The chemical composition of samples comprised the determination of crude protein (N × 6.25) (method 954.01), total lipid (method 920.39), moisture content (method 934.01), and ash content (method 942.05) using the official procedures of the AOAC [46]. In addition, the total dietary fiber (TDF) content was determined according to the method reported by Patiño-Rodríguez et al. [47] based on the AACC method 32-05.01 [48]. This methodology was conducted in triplicate.

#### 3.3.2. Apparent Amylose Content

Apparent amylose content was determined by the method reported by Espinosa-Solis et al. [49]. Samples were defatted with a Soxhlet apparatus using a solution of methanol 85% (*v*/*v*) for 24 h. The samples were washed with ethanol and recovered by filtration. The affinities of defatted starches to the iodine reagent were measured using an automatic potentiometer (702 SM Tirino, Metrohm, Herisau, Switzerland). The apparent amylose content was determined by dividing the iodine affinity of defatted starches by 20%, as reported by Takeda and Hizukuri [50]. The analysis was performed in triplicate for each starch sample.

#### 3.3.3. Color Attributes

The color of native and modified samples was evaluated according to the methodology reported by Flores-Peña et al. [51] using a Minolta CR-300 colorimeter (Minolta, Co., Ltd., Osaka, Japan). The equipment was calibrated with a white standard. The readings were taken from random points on the surface of the samples and reported in the CIELAB system. The hue angle (°h) and chromaticity (C*) were determined using the CIELAB scale. Color measurements were performed five times for each sample.

### 3.4. Thermal Properties

The thermal properties of the samples were determined with a differential scanning calorimeter (DSC) (Perkin Elmer, model DSC 4000, STA 6000) according to the procedure described by Paredes-Lopez et al. [52]. Two mg of starch was weighed in an aluminum pan and mixed with 7 μL of distilled water. The pans were hermetically sealed and left to rest for 1 h at room temperature for moisture balance. Afterward, the pans were heated from 20 to 120 °C at a heating speed of 10 °C/min. Determinations were made in triplicate for each sample.

### 3.5. Granule Morphology

The morphology of the starch granules was evaluated by scanning electron microscopy (SEM). A JEOL microscope was used (model JSM-6010A, Tokyo, Japan). The powders samples were attached to a double-adhesion graphite tape in a metal sample holder and covered with a layer of gold to make it conductive. Finally, under the microscope, images were taken at an acceleration potential of 10 kV.

### 3.6. In Vitro Digestibility of Native and Autoclaved Samples

#### 3.6.1. Total Starch

Total starch (TS) content was determined under the method reported by Goñi et al. [53]. For this, 50 mg of sample was dispersed in a solution of KOH 2 M to gelatinize the starch. The mixture was left for 30 min and subsequently incubated at 60 °C for 45 min, at a pH of 4.75, with an amyloglucosidase enzyme solution (Marca Roché, No. 102 857, Roche Diagnostics, IN, USA). After that time, the glucose content released was determined by the glucose oxidase–peroxidase test (GOPOD) (SERA-PAK^®^ Plus, Bayer de Mexico, S.A. de C.V.). The TS content was calculated as glucose (mg) × 0.9, using potato starch as a reference. The analysis was performed in triplicate for each sample.

#### 3.6.2. Available Starch

Available starch (AS) was determined according to the method reported by Holm et al. [54]. Five hundred mg sample was combined with 20 mL of distilled water, the mixture was stirred for 10 min, and then 100 µL of α-amylase (Termamyl) was added. The mixture was boiled for 20 min and stirred every 5 min until 20 min was completed, then left to rest until the sample cooled down to 30–40 °C and transferred to a 100 mL volumetric flask. In a glass tube, 1 mL of sodium acetate buffer (pH 4.75), 25 µL of amyloglucosidase, and 500 µL of the sample were placed and incubated for 30 min at 60 °C with constant agitation. The content of the tubes was transferred to a 10 mL volumetric flask, and 50 µL of the sample was taken to determine glucose released by enzyme digestion, using the GOD/POD test, reading the optical densities of the samples at 510 nm.

#### 3.6.3. Resistant Starch

For the determination of resistant starch (RS) content in the samples, the methodology described by Goñi et al. was performed [53]. This technique quantified the indigestible starch fraction using the glucose freed by enzymatic digestion, using the GOD/POD test by reading the optical densities of the samples at 510 nm.

#### 3.6.4. Retrograded Resistant Starch

The determination of retrograded resistant starch (RRS) followed the methodology described by Saura-Calixto et al. [55]. One hundred mg of sample was incubated with thermostable α-amylase, protease, and amyloglucosidase. Afterward, the solution was centrifuged for 15 min at 3000× *g*, discarding the supernatant, and the pellet was washed with ethanol and acetone. Finally, the pellet was re-suspended with 2 M KOH and incubated with amyloglucosidase, and centrifuged for 15 min at 3000× *g*, and the supernatant was collected. Fifty µL of supernatant was taken to determine glucose content using the GOD/POD test, reading the optical densities of the samples at 510 nm.

### 3.7. The Molar Mass of Amylose

The weight-average molar mass of amylose was determined by HPSEC-RI, for this purpose, a calibration line was obtained using pullulan standards of various molar masses (1.6 × 10^6^, 3.8 × 10^5^, 1.8 × 10^5^, 1.0 × 10^5^, 4.8 × 10^4^, and 1.2 × 10^4^ g/mol). Amylose was isolated from native and modified malanga flour and corn starch following Torruco-Uco et al. [56]. Afterward, the amylose was solubilized with 50 s microwave heating and filtered through a 5 µm nylon syringe filter. The solution was injected into an HPLC equipment (Agilent 1100 series; Agilent Technology, Deutschland GmbH Waldbronn, Germany) to separate amylose molecules, a GPC-SEC column was used (PL aquagel-OH, 8 µm column, 7.5 mm ID × 300 mm; Agilent Technologies Deutschland GmbH Waldbronn, Alemania). The column and the detector were maintained at 30 °C. The eluent was HPLC-grade water, carefully degassed and filtered before use through Durapore GV (0.2 μm) membranes. The flow rate was 1.0 mL/min. The data analysis was realized using the GPC software of Agilent (Agilent Technologies Deutschland GmbH Waldbronn, Germany). The carbohydrate concentration of the supernatant solution after filtration was measured by the sulfuric acid–phenol colorimetric method. The procedure was carried out at least five times for each sample.

### 3.8. Fourier Transform Infrared (FTIR) Spectroscopy

The FTIR analysis of the samples was performed according to the methodology recently reported by Tirado-Gallegos et al. [57] using a Spectrum Two infrared spectrophotometer (Perkin Elmer Inc., Waltham, MA, USA). The procedure consisted of putting each of the samples in Ziploc™ bags (approximately 100 mg of each sample). Subsequently, each of the bags containing the sample was homogenized, and from there, a small amount was taken (≈5 mg). Samples were placed in the sample holder for ATR, and utilizing a punch, a pressure of 100 ± 1 N was exerted on the sample. The vibrational transition frequencies were reported in transmittance (%) according to the wavenumber (cm^−1^) within the mid-infrared. An average of 34 sweeps per sample was recorded with a resolution of 4 cm^−1^ in the region of 400 to 4000 cm^−1^. The molecular spectral data of samples were collected and corrected with the air background and further analyzed with Spectrum Two software version 10.4. The analysis was performed in triplicate for each sample. Spectral analysis of each of the samples was selected. Therefore, the reported spectra are representative of each of the individual samples.

### 3.9. Statistical Analysis

The experiments were conducted using a completely randomized design. A one-way variance analysis (ANOVA, *p* ≤ 0.05) was used to apply the SigmaStat statistical program, version 2.03 [58]. When significant differences were found, Tukey´s test was applied (*p* ≤ 0.05) [59].

## 4. Conclusions

The physicochemical, morphological, and FTIR analyses showed, in the malanga flour, the presence of minor components other than the starch. These components influenced the color and thermal properties of the malanga flour and its respective autoclave samples. The autoclaving treatment modified the physicochemical, morphological, and thermal properties, which were similar in both of the native samples (malanga flour and commercial corn starch). The autoclaving treatment increased the RS and RRS contents in the malanga flour. These results potentiate malanga flour by obtaining food with the content of dietary fiber and mineral salts practically equal to that of native flour, and with a greater presence of RS, proteins, and lipids than that observed in native and resistant starch from a commercial source, such as corn starch. Malanga flour can be considered as a feasible raw material for obtaining resistant starch type III (RS3), with physicochemical, morphological, and thermal properties similar to RS3 obtained from commercial corn starch.

## Figures and Tables

**Table 1:** Chemical composition of malanga flour, corn starch and their respective resistant starches *.

Composition (%)	Sample ^1^			
	MF	AMF	CS	ACS
Moisture	15.65 ± 0.51 ^a^	9.38 ± 0.94 ^b^	14.36 ± 0.80 ^a^	7.13 ± 0.71 ^c^
Ash	6.31 ± 1.07 ^a^	6.60 ± 0.94 ^a^	5.69 ± 1.20 ^a^	1.81 ± 0.64 ^b^
Protein ^2^	2.79 ± 0.95 ^a^	1.06 ± 0.27 ^b^	0.68 ± 0.05 ^c^	0.37 ± 0.03 ^d^
Fat	1.08 ± 0.21 ^a^	0.72 ± 0.21 ^a^	0.11 ± 0.03 ^b^	0.10 ± 0.04 ^b^
Carbohydrate ^3^	74.17 ± 0.38 ^c^	82.24 ± 0.68 ^b^	79.16 ± 0.85 ^c^	90.59 ± 0.15 ^a^
Total dietary fiber ^4^	1.58 ± 0.13 ^a^	1.03 ± 0.19 ^b^	0.36 ± 0.08 ^c^	0.29 ± 0.05 ^d^
Yield	Nd	89.3 ± 0.31 ^a^	Nd	89.5 ± 0.37 ^a^

* Values represent the average of three repetitions ± standard error. Different letters in each row are significantly different (*p* < 0.05). ^1^ Samples are MF = malanga flour; AMF = autoclaved malanga flour; CS = corn starch; ACS = autoclaved corn starch. ^2^ Quantification by Kjeldahl method, conversion factor N_2_ × 6.25. ^3^ Obtained by difference (100% − [% moisture + % ash + % protein + % fat]). ^4^ Dry basis, Nd: no determinate.

**Table 2:** Results of apparent amylose content and average amylose molecular weight of samples *.

Sample ^1^	Mw × 10^3^ (g/mol) ^2^	Apparent Amylose (%)
MF	93.12 ± 2.51 ^b^	28.36 ± 1.65 ^a^
AMF	84.70 ± 1.80 ^c^	23.84 ± 1.18 ^b^
CS	110.35 ± 8.60 ^a^	27.35 ± 0.98 ^a^
ACS	85.10 ± 3.75 ^c^	22.18 ± 1.27 ^b^

* Values represent the average of three repetitions ± standard error. Different letters in each column are significantly different (*p* < 0.05). ^1^ Samples are MF = malanga flour; AMF = autoclaved malanga flour; CS = corn starch; ACS = autoclaved corn starch. ^2^ Weight average molar mass.

**Table 3:** Color parameters of malanga flour, corn starch, and their respective autoclaved powders *.

Parameter	Sample ^1^			
	MF	AMF	CS	ACS
L*	91.50 ± 0.08 ^c^	78.12 ± 0.11 ^d^	100.02 ± 0.02 ^a^	97.90 ± 0.06 ^b^
C*	4.49 ± 0.02 ^b^	14.26 ± 0.17 ^a^	4.91 ± 0.03 ^b^	2.79 ± 0.00 ^c^
°h	38.58 ± 0.30 ^d^	64.98 ± 0.09 ^c^	106.52 ± 0.49 ^a^	100.24 ± 0.39 ^b^

* Values represent the average of five repetitions ± standard error. Different letters in each row are significantly different (*p* < 0.05). ^1^ Samples are MF = malanga flour; AMF = autoclaved malanga flour; CS = corn starch; ACS = autoclaved corn starch.

**Table 4:** Thermal variables of gelatinization of native and autoclaved malanga flour and corn starch samples.

Sample ^1^	Thermal Variables ^2^			
	T_o_ (°C)	T_p_ (°C)	T_c_ (°C)	ΔH (J/g)
MF	75.14 ± 0.13 ^b^	84.56 ± 0.13 ^b^	92.03 ± 0.27 ^b^	12.49 ± 0.46 ^a^
AMF	86.13 ± 0.80 ^a^	99.17 ± 0.81 ^a^	112.31 ± 1.18 ^a^	9.36 ± 0.61 ^b^
CS	70.01 ± 0.23 ^c^	74.01 ± 0.02 ^c^	79.81 ± 0.06 ^c^	7.47 ± 0.14 ^c^
ACS	85.36 ± 0.72 ^a^	98.14 ± 0.65 ^a^	110.11 ± 0.84 ^a^	8.11 ± 0.50 ^b^

* Values represent the average of three repetitions ± standard error. Different letters in each column are significantly different (*p* < 0.05). ^1^ Samples are MF = malanga flour; AMF = autoclaved malanga flour; CS = corn starch; ACS = autoclaved corn starch. ^2^ Thermal variables are onset temperature (T_o_), peak temperature (T_p_), conclusion temperature (T_c_) and enthalpy change (∆H).

## Data Availability

Data are contained within the article.

## Sample Availability

Samples are available from the authors.
